# Persistent trigeminal artery in a patient with posterior circulation stroke treated with rt-PA: case report

**DOI:** 10.1186/s12883-019-1492-2

**Published:** 2019-10-27

**Authors:** Axel Ferreira, Paulo S. Coelho, Vítor Tedim Cruz

**Affiliations:** 1 Neurology Department, Hospital Pedro Hispano, ULS Matosinhos, Porto, Portugal; 2 EPIUnit, Institute of Public Health University of Porto, Porto, Portugal

**Keywords:** Posterior trigeminal artery, Stroke, Vertebrobasilar insufficiency, Saltzman classification, CT angiography

## Abstract

**Background:**

A persistent trigeminal artery (PTA) is a non-involuted embryonic vessel that connects the cavernous part of the internal carotid artery with the posterior circulation. In the adult it is associated with multiple pathological conditions including trigeminal neuralgia, ophthalmoplegia, hypopituitarism, intracavernous fistula, brain aneurysms and posterior circulation strokes. The latter may occur through steal phenomena or thrombosis in the anterior circulation. PTA associated vertebrobasilar hypoplasia has yet to be associated to TIA like events, however, in the reported case, that seems to be the case with reported vertigo being probably linked to vertebrobasilar insufficiency.

**Case report:**

We present a case of an 82-year-old man with sudden onset neurological deficits, including left hemiparesis with crural predominance, vertical nystagmus, right internuclear ophthalmoplegia, dysarthria and dysmetria on the left arm. CT angiography disclosed basilar artery hypoplasia in the proximal two thirds and a persistent trigeminal artery. He was diagnosed with acute ischemic stroke. He was submitted to rt-PA with partial reversion of deficits.

**Conclusion:**

The ischemic events related to PTA remain a rare cause of stroke with specific pathophysiological mechanisms and implications. They may occur through steal phenomena or thrombosis in the anterior circulation. Upon literature review, in the described case both mechanisms seem possible, however the transient episodes of vertigo could have been the first sign of vertebrobasilar insufficiency.

## Background

The trigeminal arteries have their origin in the embryonic vessels that connect the cavernous portion of the developing internal carotid arteries (ICA) and the paired longitudinal neural arteries that will later form the basilar artery (BA) [1, 2]. While the trigeminal artery usually involutes after the development of the posterior communicating artery (PcoA) there are cases, for reasons still unclear, where it remains persistent [2]. The PTA is the most common persistent embryonic carotid–basilar anastomosis [1]. Prevalence varies from 0.12 to 1% in studies using magnetic resonance angiography imaging or classical angiography [3–8]. In this paper we will briefly explain what is a PTA and to what pathologies it can be associated, we will review the meaning of a PTA in a posterior circulation stroke through a clinical case and we will present a table that summarizes the PTA related strokes reported in literature.

**Fig. 1:**
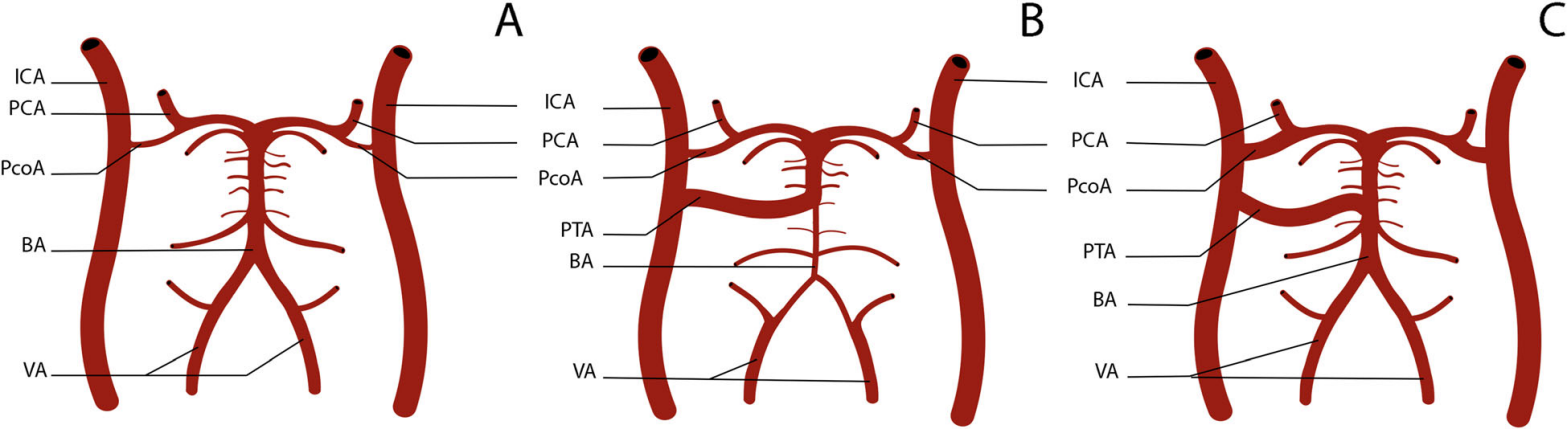
– Schematic representation of the vertebrobasilar system, **a**) without PTA, **b**) with Saltazman type 1 PTA, showing BA hypoplasia proximal to the PTA, **c**) with Saltazman type 2 PTA, showing the PCAs predominantly supplied by the PcoA. BA – basilar artery, ICA – internal carotid artery, PCA – posterior cerebral artery, PcoA – posterior communicating artery, PTA – persistent trigeminal artery, VA – vertebral artery

The different variations of the PTA can be cataloged using the Saltzman classification (Fig. 1), [1, 9, 10]. In Saltzman type 1, also called fetal PTA, the PTA insertion in the BA is distal to the anterior inferior cerebellar artery (AICA) and proximal to the superior cerebellar artery (SCA) and, in some cases, the BA proximal to the insertion of the PTA may be hypoplastic and the PcoA of the same side may be absent. In the Saltzman type 2 there is usually no hypoplasia of the BA, the PTA inserts proximally to the SCA, supplying them, and the PCAs are predominantly supplied by the PcoA. In the Saltzman type 3 variant, the PTA inserts directly into one of the cerebellar arteries, without having an anastomosis with the BA. In the case of Slatzman type 3 there are 3 variants: the type 3a variant that terminates in SCA; the type 3b variant, and the the most common one, that terminates into AICA; and type 3c variant that terminates into posterior inferior cerebellar artery (PICA) [1, 2]. In one study of 4.650 patients that underwent brain MRA, the prevalence of each type using the Saltzman classification was as follows: type I, 24%; type II, 16%; type III, 60% [7].

The PTA is linked to several pathologies, including vascular nerve compression syndromes, like trigeminal neuralgia (more prevalent in patients with PTA) [11, 12] or ophthalmoplegia due to oculomotor or abducens palsy [12–14]. It is also linked to hypopituitarism due to compression of the pituitary stalk [14, 15], spontaneous or traumatic intracavernous fistula [16] and brain aneurysms. Although there is no agreement that brain aneurysms are more prevalent in patients with PTA [4, 7, 17], the PTA itself is prone to aneurysms due to its bifurcation [1]. Finally, the PTA is also related to ischemic stroke, although, to our knowledge it has never been linked to TIA by vertebrobasilar insufficiency [18–30]. We present in Table 1 a structured summary of the PTA related stroke cases reported in literature.

**Table 1:** Summary description of PTA related stroke episodes found upon literature review, cases are ordered according to patient’s age at symptom onset

Identification	Symptoms	Territory Confirmed by neuroimaging	Arteries involved and saltzman classification of PTA	Treatment	Patient outcome
42yo F (2010) [18]	Headaches, visual disturbances, right-sided numbness progressing to dysarthria and right-sided motor deficit with central facial palsy	Left anterolateral pontine infarction	Internal carotid artery (ICA) and PTA occlusion Saltzman type 1	Heparine	Residual right motor deficit
47yo M (1992) [19]	Right hemiparesis and numbness of the left face	Ventral pontine lacunar infarct	Hypoplasia of the VAs and VB Saltzman type 2	Urokinase	Hemiparetic gate
54yo M (2006) [20]	Aphasia, dysarthria and right hemiparesis	Left middle cerebral artery and posterior cerebral artery territories	Embolization to the posterior circulation through the PTA Saltzman type 1	None	No deficits
55yo M (2010) [21]	Dysarthria and right hemiparesis (grade 4/5)	Left ventral hemipontine infarction	Ipsilateral tortuous primitive trigeminal artery Salytzman type 1	Unknown	Unknown
56yo M (2014) [22]	Transient diplopia and right-sided numbness which resolved in 10–15 min, 2 days later horizontal nystagmus and diplopia, right hemisensory loss to light touch and pinprick, tongue deviation to the right, decreased fine motor function in the right hand and slow right finger-to-nose performance	Left ventral pons and left superior cerebellar peduncle	Thrombosis of a persistent left trigeminal artery Saltzman type 1	None	Unknown
58yo M (2016) [23]	Retrograde and anterograde amnesia, superior homonymous quadrantanopias, and could not identify colors	Bilateral occipital infarcts involving the parahippocampal and lingual gyri	Hypoplastic vertebrobasilar circulation, with a persistent right trigeminal artery supplying the rostral basilar artery Saltzman type 1	Unknown	Unknown
62yo F (2006) [24]	Transient ill-defined visual disturbance and a mild right-sided hemiparesis	Left mesencephalic	Right high-grade ICA stenosis (80%) hypoplastic vertebral arteries, right PTA Saltzman type 1	ICA endarterectomy	No deficits
63yo M (1998) [25]	1. Left facial palsy, right hemiparesis and left medial longitudinal fasciculus syndrome. 2. Hemiparesis and hemihypesthesia including pain and touch on the left side.	1. Left basal part of the pons 2. Right basal part of the pons	Basilar artery middle portion stenosis (80%) Saltzman type 1	1. None 2. Urokinase	1. Slight left facial palsy and right hemiparesis 2. No new deficits
67yo M (2015) [26]	Sudden loss of consciousness and quadriplegia	Right cerebral hemisphere	Right middle cerebral artery (MCA) and BA occlusion Saltzman type 1	Thrombectomy of the MCA and of the BA through the PTA	Lucid and with moderate left hemiparesis
67yo M (2015) [27]	Sudden loss of consciousness and left hemiparesis	None	Right ICA occlusion with PTA, BA, posterior cerebral artery and superior cerebellar artery hypoperfusion Saltzman type 1	rt-PA and thrombectomy through the ICA and PTA	Mild facial and left hand paresis
70yo M (2010) [28]	Dysarthria and right hemiparesis	Left pontine infarction	Embolic occlusion of the PTA Saltzman type 1	rt-PA	No deficits
73yo M (2014) [29]	Weakness of the lower extremities, and blindness of the left eye	Multiple infarcts in the anterior and posterior circulation (cardioembolisms)	Persistent primitive trigeminal artery, basilar artery hypoplasia Saltzman type 1	Unknown	Unknown
76yo M (2016) [30]	Mild dysmetria and intentional tremor affecting the right arm, tandem gait ataxia, and right-sided hemianopia, preceded several weeks by intermittent vertigo	Border-zone territory between the MCA and the posterior cerebral artery (PCA)	Left ICA stenosis (75%) and left PTA, BA hypoplasia below the PTA Saltzman type 1	ICA endarterectomy	Resolution of the intermittent vertigo
80yo F (2010) [29]	Dysphasia and dysarthria with dizziness and cough	Right pontine infarct	PTA between left vertebral artery and ipsilateral external carotid artery, right vertebral artery hypoplasia Saltzman type 2	Unknown	Unknown

## Case presentation

An 82-year-old man, presented to the emergency department, on February 2018, after sudden onset of left hemiparesis and vertigo. Symptoms started during a period of greater physical effort, upon participation in a *zumba* class. His past medical history included transient episodes of vertigo during exercise in the previous months, an anterior circulation right hemispheric stroke in 2015, that left no sequels, and hypertension controlled with a combination of 10 mg lisinopril and 2.5 mg amlodipine. The neurologic examination revealed mild left hemiparesis with facial involvement and crural predominance, vertical nystagmus, right internuclear ophthalmoplegia, dysarthria and dysmetria on the left arm. The total National Institutes of Health Stroke Scale (NIHSS) score was 6.

**Fig. 2:**
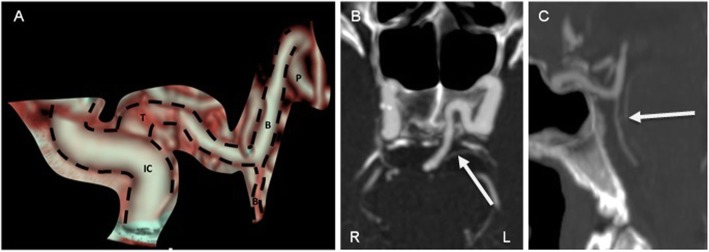
– Head CT scan showing the PTA, **a**) volume rendering technique showing PTA anastomosing the left ICA with de the terminal portion of the BA, **b**) arrow indicating PTA originating from the left ICA, **c**) arrow showing hypoplastic BA proximal to the anastomosis. IC – internal carotid artery, T – trigeminal artery, B – basilar artery, P – posterior cerebral artery

The plain brain CT scan was normal and a CT angiography showed hypoplasia of both vertebral arteries, the left terminating as the PICA, while the right gave origin to the BA. The BA had a filiform aspect in its proximal two thirds, having a normal caliber in the distal remaining third, after receiving a communicating artery from the cavernous segment of the left internal carotid artery, a PTA (Fig. 2). No abrupt stop of flow was identified. Echocardiogram showed severe dilation of the left auricle, as well as mild dilation of the right auricle and a 35 mm dilation of the proximal portion of the ascending aorta. Electrocardiogram was normal and a 48 h cardiac telemetry monitoring didn’t show any periods of arrhythmia. Lipid profile showed borderline high low density lipoprotein level, at 139 mg/dL.

The patient underwent treatment with recombinant tissue plasminogen activator (rt-PA) at two hours of symptom onset, this decision was based on the clinical presentation and in the absence of hemorrhage in the CT scan. Shortly after treatment the NIHSS score was 2, maintaining a slight paresis of the left leg and dysmetria on the left arm. Months later, the patient showed no neurologic sequelae.

## Discussion and conclusions

PTA can be associated with many different vascular events. Patients with PTA and vertebrobasilar hypoplasia have a tendency to have a decreased vascular supply to the posterior fossa [19], this fact renders them susceptible to ischemic events. In this context, in the case of a stenosed carotid artery, a steal phenomenon can occur, and this can lead either to vertebrobasilar insufficiency or hemodynamic brain stem infarction [31]. In the case of BA occlusion however, if the occlusion is proximal to the insertion of the PTA, the PTA can have a protective effect in the distal territory [25]. In the case of an anterior circulation thrombus, the PTA can lead to migration of the thrombus to the BA, possibly causing a posterior circulation stroke [20, 24]. Finally, in BA hypoplasia, PTA also opens the possibility for thrombectomy in the posterior circulation [26, 27].

Despite all the variety of ischemic events related with PTA, thrombosis of this vessel remains a rare phenomenon, although there is a case report of PTA thrombosis related with internal carotid dissection [18].

Our patient had a congenital Saltzman type 1 PTA variant and presented with symptoms suggestive of acute brainstem infarction that partially resolved after the administration of rt-PA. The clinical manifestations are compatible with an ischemic lesion of the basis pons, causing an ataxic hemiparesis syndrome, and extending to the tegmental region, affecting the longitudinal medial fasciculus. This suggests that the most affected territory was dependent from the small perforating vessels of the BA. Furthermore, the patient was exposed to extenuating exercise when the symptoms started, this favors the possibility that the event was due to a steal phenomenon, but the fact that the patient had no great artery disease and the fact that the patient improved after rt-PA suggests that the mechanism could also have been thromboembolic. Upon literature review both mechanisms seem possible. We would also like to emphasize that the reported episodes of vertigo probably corresponded to previous transient ischemic attacks (TIA) in the same area, possibly being the first sign of vertebrobasilar insufficiency. To our knowledge this is the first case study to point out the possibility of having several transient episodes (in this case of vertigo) compatible with vertebrobasilar insufficiency as a premonitory sign of PTA related stroke.

## Data Availability

Not applicable.

## Abbreviations

AICA: Anterior inferior cerebellar artery
BA: Basilar artery
ICA: Internal carotid artery
NIHSS: National Institutes of Health Stroke Scale PcoA - posterior communicating artery
PTA: Persistent trigeminal artery
rt-PA: Recombinant tissue plasminogen activator
SCA: Superior cerebellar artery
TIA: Trasient ischemic attacks
VA: Vertebral artery
